# The emerging role of copper in depression

**DOI:** 10.3389/fnins.2023.1230404

**Published:** 2023-08-07

**Authors:** Jinhua Chen, Wenping Song, Wenzhou Zhang

**Affiliations:** Department of Pharmacy, Affiliated Cancer Hospital of Zhengzhou University and Henan Cancer Hospital, Henan Engineering Research Center for Tumor Precision Medicine and Comprehensive Evaluation, Henan Provincial Key Laboratory of Anticancer Drug Research, Zhengzhou, China

**Keywords:** copper, depression, homeostasis, oxidative stress, inflammatory response, dietary supplementation

## Abstract

Copper (Cu) is an essential trace element in the brain and serves as an important cofactor for numerous enzymes involved in a wide range of biochemical processes including neurobehavioral, mitochondrial respiration, and antioxidant effects. Recent studies have demonstrated that copper dyshomeostasis is tightly associated with the development of depression by inducing oxidative stress and inflammatory responses. However, these findings have remained controversial so far. Cumulative studies have shown a positive association, while some other studies showed no association and even a negative association between serum/plasma copper level and depression. Based on these conflicted results, the association was speculated to be due to the clinical features of the population, stages of the disease, severity of copper excess, and types of specimens detected in these studies. In addition, there was an inverse association between dietary copper intake and depression. Furthermore, increasing copper intake could influence dietary zinc and iron intake to prevent and treat depression. Thus, copper supplementation may be a good measure to manage depression. This review provided a deeper understanding of the potential applicability of copper in the prevention and treatment of depression.

## 1. Introduction

Depression is one of the leading mental disorders, and the number of people with depression has reached approximately 280 million worldwide from World Health Organization (WHO) statistics, making it a major contributor to the overall global burden of diseases (World Health Organization, 2023a,b). Given the deleterious effect of depression on human health, WHO’s Mental Health Gap Action Programme (mhGAP) has listed it as a priority condition in its Mental health action plan 2013–2030 (Institute of Health Metrics and Evaluation, 2023). There are many etiologies involved in depression including social, psychological, and biological factors (Ferrari et al., 2016). It is characterized by several symptoms such as depressed mood, hopelessness about the future, and thoughts of dying or suicide (World Health Organization, 2023a,b). Depression can be categorized as mild, moderate, and severe on the basis of the number and severity of symptoms, as well as the impact on the individual’s functioning. Depending on the severity and pattern of depressive episodes, different treatments are recommended, including psychological treatment and antidepressant medications (Shusharina et al., 2023), but a significant proportion of people who received treatment still fail to achieve remission (Mauskopf et al., 2009; Moriarty et al., 2020) and more than 75% of people in low and middle-income countries do not receive any treatment (Evans-Lacko et al., 2018). Hence, it is imperative to look for new risk factors and effective treatments to prevent and treat depression.

Copper (Cu) is an essential trace element and the third most abundant trace metal after iron and zinc in the human body (Barceloux, 1999). It is almost entirely absorbed in the gastrointestinal tract, stored in the liver, and eliminated through biliary excretion (Halliwell and Gutteridge, 1984). It is a vital cofactor of numerous important enzymes, such as dopamine monooxygenase, cytochrome oxidase, and the free radical scavenger superoxide dismutase (Uauy et al., 1998; Turnlund, 2000), and is involved in a wide range of biochemical processes including neurobehavioral, mitochondrial respiration, and antioxidant effects (Uriu-Adams and Keen, 2005). The roles of copper in mental diseases have attracted the attention of researchers due to its high levels in the brain (Rihel, 2018). An imbalance in copper levels in the brain has been reported to be associated with many neuropathic diseases, such as depression, Alzheimer’s disease, Menkes disease, and Wilson’s disease (An et al., 2022). Several studies have explored the association between copper levels in the human body and depression, but their conclusions remain controversial. Cumulative studies have shown a positive association between serum/plasma copper levels and depression (Russo, 2011; Habibi et al., 2017; Islam et al., 2018; Ni et al., 2018; Ullas Kamath et al., 2019; Wu et al., 2022), while some other studies showed that there were no associations (Styczeń et al., 2016; Siwek et al., 2017) and even negative associations between serum/plasma copper level and depression (Styczeń et al., 2016; Li et al., 2018; Twayej et al., 2020; Ding and Zhang, 2022). Given these conflicting results, we focused on the role of copper in depression and its underlying mechanisms in this review, aiming to provide a better understanding of its potential applicability in preventing and treating depression.

## 2. Regulation of copper homeostasis

**Figure 1:**
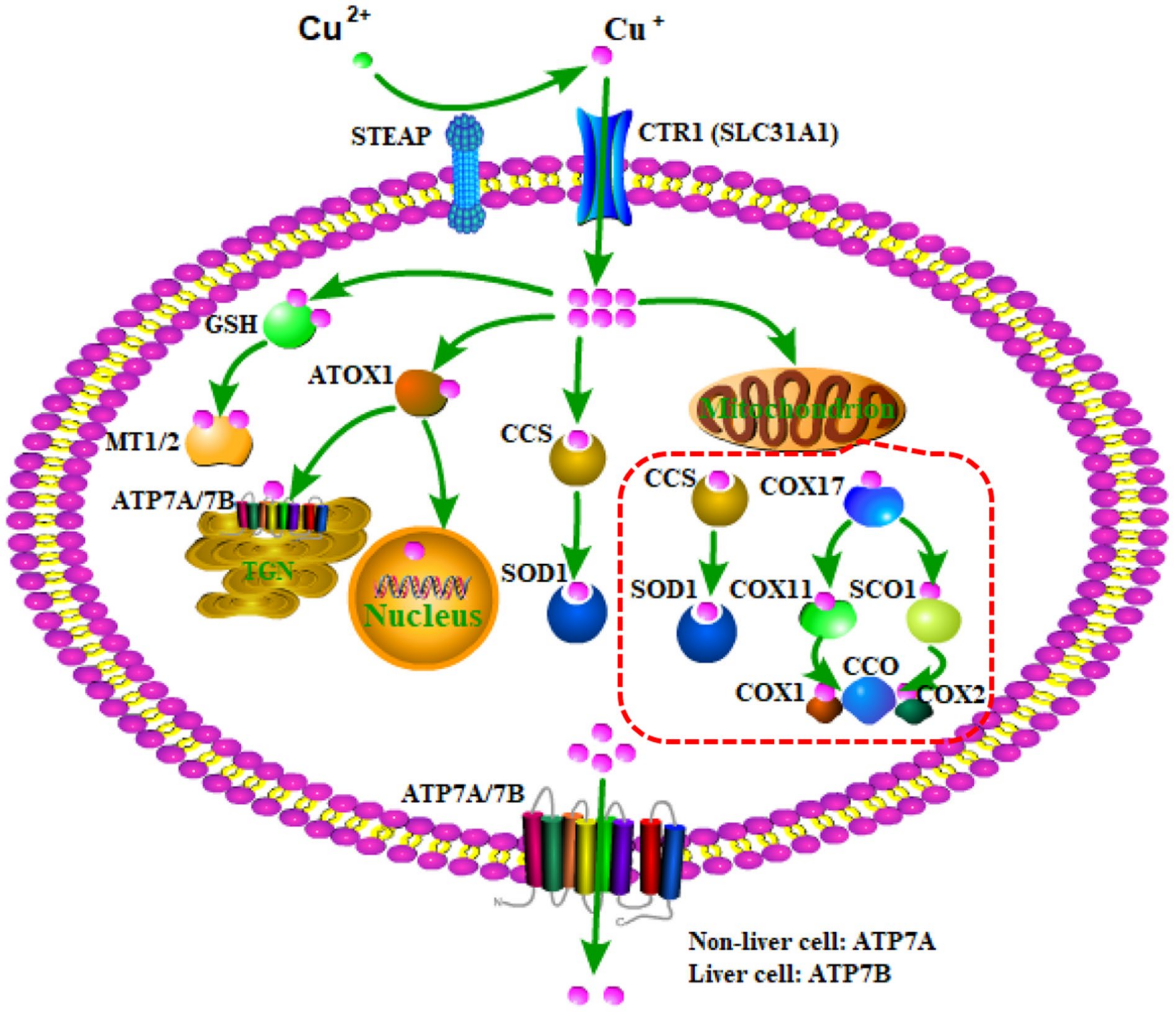
The regulation process of copper homeostasis. SETAP, metalloreductase six-transmembrane epithelial antigen of the prostate; CTR1, copper transporter 1; SLC31A1, solute carrier family 31 member 1; GSH, glutathione; MT1/2, metallothionein 1/2; ATOX1, the chaperone antioxidant-1; CCS, copper chaperone for superoxide dismutase; SOD1, superoxide dismutase 1; COX17, chaperone cytochrome oxidase 17; SCO1, chaperone synthesis of cytochrome oxidase 1; CCO, cytochrome C oxidase; COX1, cytochrome C oxidase (CCO) I; COX2, cytochrome C oxidase (CCO) II.

Copper homeostasis, namely, the dynamic balance in copper levels, is a tightly regulated process by various key molecules, including copper chaperones, transmembrane transporters, and transcriptional regulators (Chen et al., 2022, 2023). These molecules cooperatively regulate the import, intracellular distribution, and export of copper to maintain homeostasis. As shown in Figure 1, copper is a redox-active metal ion that exists in two oxidation states: Cu^+^ and Cu^2+^ (Ge et al., 2022). Extracellular Cu^2+^ binding to ceruloplasmin is reduced by the metalloreductase six-transmembrane epithelial antigen of the prostate (SETAP) to Cu^+^, and copper transporter 1 (CTR1) (also known as solute carrier family 31 member 1, SLC31A1) transports Cu^+^ into cells (Ohgami et al., 2006). Once it enters the cytoplasm, a part of Cu^+^ binds to glutathione (GSH) and is delivered to metallothionein 1/2 (MT1/2) to be restored, and other parts of Cu^+^ are either transferred to the nucleus or ATP7A/7B located in the trans-Golgi network (TGN) by the chaperone antioxidant-1(ATOX1) to facilitate the synthesis of cuproenzymes (Lutsenko et al., 2007) or delivered to superoxide dismutase 1 (SOD1) in the cytoplasm and mitochondrial intermembrane space by a copper chaperone for superoxide dismutase (CCS) to detoxify reactive oxygen species (ROS). In addition, Cu^+^ in the cytoplasm can be transported to the mitochondrial intermembrane space, in which Cu^+^ binds to chaperone cytochrome oxidase 17 (COX17) and is delivered to either the chaperone synthesis of cytochrome oxidase 1 (SCO1) or COX11, ultimately delivering to the cytochrome C oxidase (CCO) I (COX1) or II (COX2) subunits to involve them in the respiratory chain (Horng et al., 2004).

Because of the alteration of physiological or pathological conditions in the human body, the cellular copper content changes, resulting in the disturbance of copper homeostasis, namely, copper excess or copper deficiency. Based on the cellular copper status, the expression of some molecules involved in copper homeostasis is regulated. For example, CTR1 and CCS are down-regulated when intracellular copper overloads and up-regulated when intracellular copper is deficient (Bertinato and L'Abbé, 2003; Liang et al., 2012). Moreover, ATP7A and ATP7B, as the major transporters for exporting cellular copper, are commonly located in the TGN. However, when intracellular copper overloads, ATP7A and ATP7B translocate from the TGN to the plasma membrane to export copper. When intracellular copper recovers to the physiological condition, ATP7A and ATP7B return to the TGN (La Fontaine and Mercer, 2007). It is important to note that the expression of ATP7A and ATP7B is tissue-specific. ATP7A is expressed in various tissues and organs, whereas ATP7B is predominantly expressed in the liver, suggesting that ATP7A, but not ATP7B, is primarily involved in the exporting of copper into the brain cell (Lutsenko et al., 2007).

## 3. The role of copper in oxidative stress and inflammation

**Figure 2:**
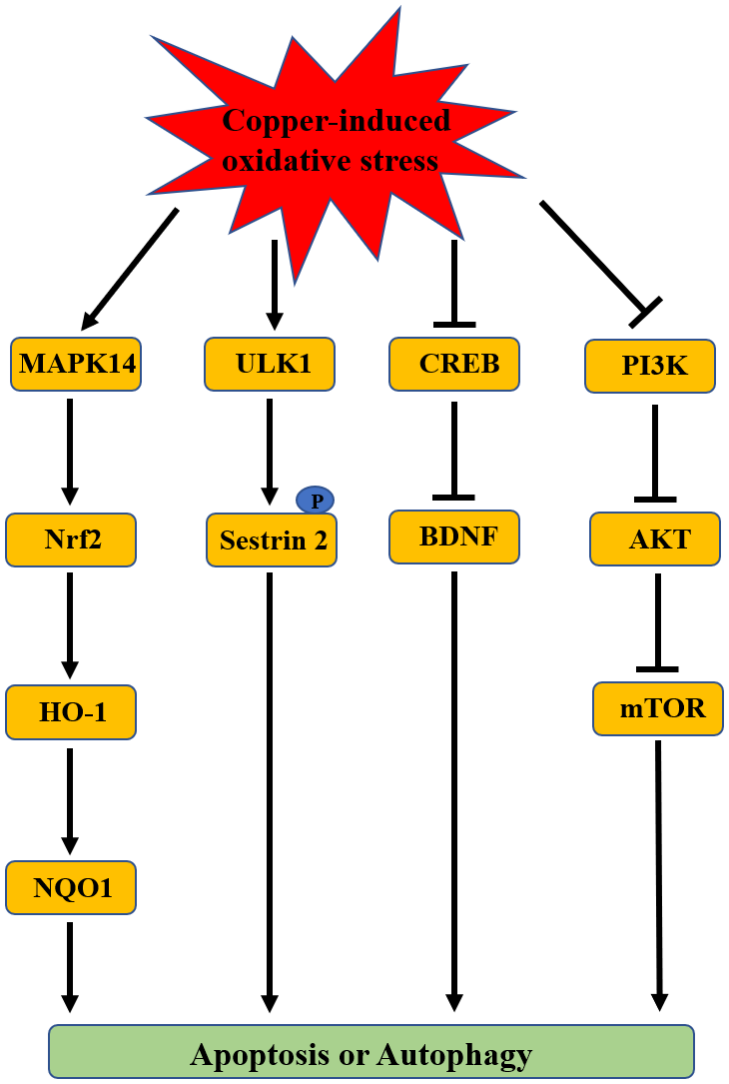
The associated signaling pathways of copper-induced oxidative stress.

Some of the molecular mechanisms underlying copper-induced depression included oxidative stress, neurotransmitter imbalance, and impaired synaptic plasticity. Among these mechanisms, oxidative stress is regarded as a mainstay because of its effect on other depression-associated mechanisms (Bhatt et al., 2020; Correia et al., 2023). Oxidative stress is a biological process caused by a disturbance between production and accumulation of ROS in cells and tissues and is responsible for some diseases such as neuropathic diseases and cancer due to its deleterious effects (Pizzino et al., 2017). The redox activity of copper induces oxidative stress via redox and Fenton reactions (Pereira et al., 2016; Ruizs et al., 2021). A positive association was observed between copper level in the serum or brain and oxidative stress (Maes et al., 1997; Frey et al., 2007; Ozcelik and Uzun, 2009; Salustri et al., 2010; Lee et al., 2013; Bajpai et al., 2014; Liu et al., 2015; Kumar et al., 2019). Copper has been revealed as a key regulator in various cell signaling pathways such as membrane receptor-associated pathways and growth factor-associated pathways (Grubman and White, 2014). The signaling pathways associated with copper-induced oxidative stress have been explored mainly based on *in vitro* cell experiments and *in vivo* animal studies. These studies demonstrated that a large amount of copper intake can result in oxidative damage by activating the antioxidant protection signals mitogen-activated protein kinase 14 (MAPK14)/the nuclear factor erythroid 2-related factor 2 (Nrf2)/heme oxygenase-1 (HO-1)/NAD(P)H:quinone oxidoreductase 1 (NQO1) pathway, inhibiting cAMP-response element binding protein (CREB)/Brain-derived neurotrophic factor (BDNF) pathway or PI3K/AKT/mTOR pathway to induce apoptosis or autophagy (Figure 2; Filomeni et al., 2011; Boilan et al., 2013; Zhong et al., 2014; Wang Y. et al., 2018; Xie et al., 2020; Zou et al., 2021; Li et al., 2022; Lu et al., 2022; Zhong et al., 2022). Kim et al. also found that the autophagy kinase ULK1 can induce the autophagic degradation of mitochondria by phosphorylating the ser-73 and ser-254 residues of Sestrin 2 under copper-induced oxidative stress conditions (Kim et al., 2020). In addition, copper can destroy the antioxidant defense system by decreasing antioxidant enzyme activities (SOD, CAT, and GSH-Px) to induce toxicity (Lai et al., 1996; West and Prohaska, 2004; Sun et al., 2018; Jian et al., 2020).

In addition to oxidative stress, accumulating evidence suggested that copper can exert toxicity, resulting in depression by triggering an inflammatory process. A number of studies revealed that high serum copper levels were associated with decreased levels of anti-inflammatory cytokines (IL4 and IL-10) and increased levels of pro-inflammatory cytokines (TNF-а, IL-6, IL-2, IL-8, and IL-1β) to trigger the pathogenesis of depression (Maes et al., 1995; Cattaneo et al., 2015; Habibi et al., 2017; Xu et al., 2021). Furthermore, there are various pathways that are involved in copper-regulated inflammation, including the nuclear factor kappa-B (NF-KB), MAPKs, JAK–STAT, and NLRP3 pathways (Deng et al., 2023). In addition, an alteration in the microbial richness and diversity of feces in Sprague–Dawley rats fed a high level of copper was associated with copper-regulated inflammatory responses (Zhang et al., 2017). Oxidative stress was deemed to be an important factor for the inflammatory response in the central nervous system (Ruiz et al., 2022). Consistent with it, increasing evidence suggested that copper-induced oxidative stress contributed to cellular inflammatory responses (Yang et al., 2020; Kouadri et al., 2021). Therefore, understanding copper-induced oxidative stress and inflammatory responses would be beneficial for the prevention and treatment of copper-related diseases.

## 4. Dysregulation of copper homeostasis and depression

Copper is abundant in the brain, especially in the cerebellum, hippocampus, basal ganglia, numerous synaptic membranes, cell bodies of cortical pyramidal, and cerebellar granular neurons (Desai and Kaler, 2008). It is regarded as an important cofactor for many enzymes that affect a variety of brain functions. Because the brain is a highly metabolizing organ, a small imbalance in copper levels may cause detrimental effects on the brain. Disturbance of copper homeostasis in the brain can cause copper excess or copper deficiency, leading to an array of diseases (Chakravarty and Chowdhury, 1984). This is because copper excess may result in injury, while copper deficiency may cause incomplete development (Sharma et al., 2014).

Copper excess has a higher incidence than copper deficiency in humans. It is toxic to many organs, especially the brain (Winge and Mehra, 1990). Multiple studies have suggested that copper levels in patients with depression were significantly higher than the control without depression (Narang et al., 1991; Butterworth, 2010; Russo, 2011; Habibi et al., 2017; Islam et al., 2018; Ullas Kamath et al., 2019; Wu et al., 2022). Additionally, copper content in the human body gradually increased in pregnant women, which may be related to the elevated levels of circulatory progesterone and estrogens; thus, it can easily cause depression (Gernand et al., 2016). In a study of 574 women aged 30–60 years with various mental and behavioral disorders, the serum copper levels were significantly higher in women with a history of post-partum depression (PPD) than in non-depressed women and depressed women without a history of PPD (Crayton and Walsh, 2007). This is consistent with a study showing that the mean level of copper in the serum was higher in pregnant Iranian adolescents with depression than in those without depression (Bahramy et al., 2020). As aforementioned, this may be because an elevated concentration of cellular copper can cause neuronal injury, resulting in depression by inducing oxidative stress and inflammatory responses. However, an inverse relationship was observed between copper serum level and depression (Styczeń et al., 2016; Li et al., 2018; Twayej et al., 2020; Ding and Zhang, 2022) even though there were no associations between copper serum level and depression in several studies (Styczeń et al., 2016; Siwek et al., 2017). Based on these conflicted results, the association was speculated to be related to the clinical features of the population, stages of the disease, severity of copper excess, and types of detected specimens in these studies (Table 1). For example, the epidemiology data suggested that the incidence of depression is about twice as common in women than in men (Kessler, 2003; World Health Organization, 2023a,b). Obesity was also a risk factor for depressive symptoms in individuals with high serum copper levels (Wu et al., 2022). However, the role of age as a risk factor for depression remains controversial. A study by Clark et al. showed that there was no association between blood copper and age, but two other studies demonstrated that they were correlated (Clark et al., 2007; Ma et al., 2014; Ni et al., 2018). Siwek et al. found that serum copper concentrations in patients with stage 1 bipolar disorder (including depression) were significantly higher than those of patients in advanced stages (2 + 3 + 4) of bipolar disorder (including depression) (Siwek et al., 2017). Moreover, a systematic review and meta-analysis of observational studies demonstrated that blood levels of copper in patients with depression were higher than those of patients without depression, while there was no difference in copper content in the hair between the two groups, suggesting that copper levels in the blood may be more sensitive to pathological changes in patients compared to those in the hair (Harvey et al., 2009; Ni et al., 2018). Thus, the level of plasma copper is currently the most widely used criterion for detecting copper content. Further systematic studies are needed to better understand the association between copper excess and depression.

**Table 1:** The influencing factors for studies on the association between copper levels and depression.

	Type of study	Countries	Results	References
Clinical features				
Sex	Epidemiology data	Worldwide	Incidence of depression was about twice as common in women than in men	World Health Organization (2023a,b)
Obesity	Cross-sectional study	America	Obesity (BMI ≥ 30 kg/m^2^) was a risk factor for people with high serum copper levels to develop depression symptoms	Wu et al. (2022)
Age	Clinical study	Canada	No relationship in people aged 30–65 years old	Clark et al. (2007)
	Clinical study	China	A relationship in children aged 3–12 years old	Ma et al. (2014)
	A systematic review and meta-analysis of observational studies	–	A relationship between blood copper and depression in people under 50 years old, but not in people over 50 years old	Ni et al. (2018)
The severity of the disease	Clinical study	Poland	Serum copper concentrations in patients with stage 1 bipolar disorder (including depression) were obviously higher than that of patients in advanced stages (2 + 3 + 4) of bipolar disorder (including depression)	Siwek et al. (2017)
Types of detected specimen	A systematic review	–	Serum copper appears to be a useful biomarker of copper status at the population level	Harvey et al. (2009)
	A systematic review and meta-analysis of observational studies	–	Blood levels of copper in patients with depression were higher than that of patients without depression, while there was no difference in copper content in the hair between the two groups	Ni et al. (2018)

Although the incidence of copper deficiency is relatively lower than that of copper excess, it cannot be ignored because it results in some diseases. In humans, Menkes syndrome is a main manifestation of copper deficiency and causes serious neurological disorders (Danks et al., 1972; Mercer, 1998). The mechanism may be that copper deficiency affects brain functioning by impairing brain mitochondrial function to damage energy metabolism (Munakata et al., 2005). In addition, increasing evidence reveals that copper deficiency results in decreased levels of plasma iron, which may be due to a decrease in the absorption and inhibition of iron released from the liver (Reeves and Demars, 2006; Pyatskowit and Prohask, 2008). Iron deficiency can induce depression, and thus copper deficiency may result in depression by decreasing the iron levels in the human body. Iron deficiency in the brain can be reversed by iron injections (Pyaskowit and Prohaska, 2008).

## 5. Copper supplementation for the prevention and treatment of depression

Increasing evidence has indicated that nutrients played a vital role in preventing and managing depression (Lai et al., 2014; Marx et al., 2017; Salehi-Abargouei et al., 2019). For example, there was an inverse relationship between dietary patterns rich in fruits and vegetables and high depressive symptoms (Xia et al., 2017; Wang C. J. et al., 2018; Cheng et al., 2019). Thus, the identification of the dietary factors involved in depression has attracted researchers’ attention in recent years. As aforementioned, copper is an essential dietary component in the human body. The adult human body contains approximately 75–100 mg of copper, and the recommended daily dosage is 0.9 mg/day in adults (Food and Nutrition Board, Institute of Medicine, 2001). Food is the primary source of daily copper intake (National Academy of Sciences, 2000). There is rich copper in various foods, such as shellfish, seeds, nuts, meats, and chocolate (Ma and Betts, 2000; Institute of Medicine (US) Panel on Micronutrients, 2001).

A number of studies have demonstrated that an imbalance in dietary copper intake contributed to the development of depression. A cross-sectional study of 14,834 US adults (7,399 men and 7,435 women) aged 18 years or older suggested that total copper intake may be an inverse association with depression, and these enrolled people given the Recommended Dietary Allowance had an obviously lower incidence of depression compared to those given less than the Recommended Dietary Allowance (Li et al., 2018). Consistent with this result, a negative association was observed between dietary copper intake and depression in two cross-sectional Japanese studies and a meta-analysis (Nakamura et al., 2019; Thi Thu Nguyen et al., 2019; Ding and Zhang, 2022). Additionally, a case–control study of 849 Korean adolescent girls aged 12–18 years also indicated that there was a high risk of depression in participants who ate more instant and processed foods and that dietary copper intake was negatively related to depression, suggesting that a reasonable dietary pattern played an important role in preventing and managing depression (Kim et al., 2015). Furthermore, an inverse association between dietary copper intake and depression was observed to be more relevant in women than in men (Thi Thu Nguyen et al., 2019; Ding and Zhang, 2022). Therefore, adequate intake of copper and reasonable dietary pattern was very important in preventing depression.

In addition to a dietary pattern that results in the dysregulation of copper intake, an imbalance of other metal ions in the human body can also influence copper to be involved in the pathogenesis of depression. In a cross-sectional study of 139 men and women aged ≥60 years in Australia, copper concentrations and copper/zinc ratios were found to be negatively associated with depressive symptoms (Mravunac et al., 2019). This is because zinc can compete with copper for absorption in the small intestine (Mravunac et al., 2019). It has been suggested that a high-iron diet might result in copper deficiency; in turn, increasing copper intake would correct many of the notable high iron-related physiological perturbations (Klevay, 2001, 2016; Reeves and Demars, 2005; Ha et al., 2017; Wang T. et al., 2018). A negative association has been observed between depression and dietary zinc and iron intake (Li et al., 2017). Thus, copper supplementation may be an effective measure to prevent and treat depression by interfering with the metabolic processes of zinc and iron.

## 6. Conclusion

In summary, copper is an essential trace element in the brain, and serves as an important cofactor for numerous enzymes involved in a wide series of biochemical processes, including neurobehavioral, mitochondrial respiration, and antioxidant effects. Thus, a trace dyshomeostasis of copper may cause serious brain diseases such as depression. Recent research has demonstrated that copper dyshomeostasis was tightly associated with the development of depression by inducing oxidative stress and inflammatory responses. However, the conclusion had remained controversial so far. Cumulative studies tended to show a positive association between serum/plasma copper level and depression, whereas some other studies showed no association and even negative associations between serum/plasma copper level and depression. Based on these conflicted results, the association was speculated to be related to the clinical features of the population, stages of the disease, severity of copper excess, and types of detected specimens in these studies. Further systematic studies are needed to better understand the association between copper excess and depression.

Furthermore, there was an inverse association between dietary copper intake and depression. Food is the primary source of daily copper intake. Thus, adequate intake of copper and a reasonable dietary pattern is very important for preventing depression. Additionally, increasing copper intake can influence dietary zinc and iron intake and is involved in the pathogenesis of depression. Therefore, copper supplementation may be a good strategy to prevent and treat depression.

## Author contributions

JC reviewed these works of literature and drafted the manuscript. WS and WZ revised the manuscript. All authors contributed to the article and approved the submitted version.

## Funding

This work was supported by the Tackling-plan Project of Henan Department of Science and Technology (No. 212102310325).

## Conflict of interest

The authors declare that the research was conducted in the absence of any commercial or financial relationships that could be construed as a potential conflict of interest.

## Publisher’s note

All claims expressed in this article are solely those of the authors and do not necessarily represent those of their affiliated organizations, or those of the publisher, the editors and the reviewers. Any product that may be evaluated in this article, or claim that may be made by its manufacturer, is not guaranteed or endorsed by the publisher.

## References

[ref1] An Y. Li S. Huang X. Chen X. Shan H. Zhang M. (2022). The role of copper homeostasis in brain disease. Int. J. Mol. Sci. 23:13850. doi: 10.3390/ijms232213850, PMID: 36430330 PMC9698384

[ref2] Bahramy P. Mohammad-Alizadeh-Charandabi S. Ramezani-Nardin F. Mirghafourvand M. (2020). Serum levels of vitamin D, calcium, magnesium, and copper, and their relations with mental health and sexual function in pregnant Iranian adolescents. Biol. Trace Elem. Res. 198, 440–448. doi: 10.1007/s12011-020-02109-8, PMID: 32166563

[ref3] Bajpai A. Verma A. K. Srivastava M. Srivastava R. (2014). Oxidative stress and major depression. J. Clin. Diagn. Res. 8:CC04-7. doi: 10.7860/JCDR/2014/10258.5292, PMID: 25653939 PMC4316245

[ref4] Barceloux D. G. (1999). Copper. J. Toxicol. Clin. Toxicol. 37, 217–230. doi: 10.1081/clt-100102421 10382557

[ref5] Bertinato J. L'Abbé M. R. (2003). Copper modulates the degradation of copper chaperone for cu,Zn superoxide dismutase by the 26S proteosome. J. Biol. Chem. 278, 35071–35078. doi: 10.1074/jbc.M302242200 12832419

[ref6] Bhatt S. Nagappa A. N. Patil C. R. (2020). Role of oxidative stress in depression. Drug Discov. Today 25, 1270–1276. doi: 10.1016/j.drudis.2020.05.001 32404275

[ref7] Boilan E. Winant V. Dumortier E. Piret J. P. Bonfitto F. Osiewacz H. D.. (2013). Role of p38MAPK and oxidative stress in copper-induced senescence. Age 35, 2255–2271. doi: 10.1007/s11357-013-9521-3, PMID: 23576095 PMC3824981

[ref8] Butterworth R. F. (2010). Metal toxicity, liver disease and neurodegeneration. Neurotox. Res. 18, 100–105. doi: 10.1007/s12640-010-9185-z, PMID: 20369313

[ref9] Cattaneo A. Macchi F. Plazzotta G. Veronica B. Bocchio-Chiavetto L. Riva M. A.. (2015). Inflammation and neuronal plasticity: a link between childhood trauma and depression pathogenesis. Front. Cell. Neurosci. 9:40. doi: 10.3389/fncel.2015.00040, PMID: 25873859 PMC4379909

[ref10] Chakravarty P. K. Chowdhury J. R. (1984). Serum copper in malignant neoplasia with special reference to the cervix uteri. J. Cancer Res. Clin. Oncol. 108, 312–315. doi: 10.1007/BF00390464, PMID: 6511804 PMC12252825

[ref11] Chen X. Cai Q. Liang R. Zhang D. Liu X. Zhang M.. (2023). Copper homeostasis and copper-induced cell death in the pathogenesis of cardiovascular disease and therapeutic strategies. Cell Death Dis. 14:105. doi: 10.1038/s41419-023-05639-w, PMID: 36774340 PMC9922317

[ref12] Chen L. Min J. Wang F. (2022). Copper homeostasis and cuproptosis in health and disease. Signal Transduct. Target. Ther. 7:378. doi: 10.1038/s41392-022-01229-y, PMID: 36414625 PMC9681860

[ref13] Cheng H. Y. Shi Y. X. Yu F. N. Zhao H. Z. Zhang J. H. Song M. (2019). Association between vegetables and fruits consumption and depressive symptoms in a middle-aged Chinese population: an observational study. Medicine 98:e15374. doi: 10.1097/MD.0000000000015374 31045783 PMC6504327

[ref14] Clark N. A. Teschke K. Rideout K. Copes R. (2007). Trace element levels in adults from the west coast of Canada and associations with age, gender, diet, activities, and levels of other trace elements. Chemosphere 70, 155–164. doi: 10.1016/j.chemosphere.2007.06.038, PMID: 17707880

[ref15] Correia A. S. Cardoso A. Vale N. (2023). Oxidative stress in depression: the link with the stress response, neuroinflammation, serotonin, neurogenesis and synaptic plasticity. Antioxidants 12:470. doi: 10.3390/antiox12020470, PMID: 36830028 PMC9951986

[ref16] Crayton J. W. Walsh W. J. (2007). Elevated serum copper levels in women with a history of post-partum depression. J. Trace Elem. Med. Biol. 21, 17–21. doi: 10.1016/j.jtemb.2006.10.001, PMID: 17317521

[ref17] Danks D. M. Campbell P. E. Stevens B. J. Mayne V. Cartwright E. (1972). Menkes's kinky hair syndrome. An inherited defect in copper absorption with widespread effects. Pediatrics 50, 188–201. doi: 10.1542/peds.50.2.188 5045349

[ref18] Deng H. Zhu S. Yang H. Cui H. Guo H. Deng J.. (2023). The dysregulation of inflammatory pathways triggered by copper exposure. Biol. Trace Elem. Res. 201, 539–548. doi: 10.1007/s12011-022-03171-0 35312958

[ref19] Desai V. Kaler S. G. (2008). Role of copper in human neurological disorders. Am. J. Clin. Nutr. 88, 855S–858S. doi: 10.1093/ajcn/88.3.855S 18779308

[ref20] Ding J. Zhang Y. (2022). Associations of dietary copper, selenium, and manganese intake with depression: a meta-analysis of observational studies. Front. Nutr. 9:854774. doi: 10.3389/fnut.2022.854774, PMID: 35369103 PMC8965358

[ref21] Evans-Lacko S. Aguilar-Gaxiola S. Al-Hamzawi A. Alonso J. Benjet C. Bruffaerts R.. (2018). Socio-economic variations in the mental health treatment gap for people with anxiety, mood, and substance use disorders: results from the WHO world mental health (WMH) surveys. Psychol. Med. 48, 1560–1571. doi: 10.1017/S0033291717003336, PMID: 29173244 PMC6878971

[ref22] Ferrari A. J. Stockings E. Khoo J. P. Erskine H. E. Degenhardt L. Vos T.. (2016). The prevalence and burden of bipolar disorder: findings from the global burden of disease study 2013. Bipolar Disord. 18, 440–450. doi: 10.1111/bdi.12423 27566286

[ref23] Filomeni G. Cardaci S. Da Costa Ferreira A. M. Rotilio G. Ciriolo M. R. (2011). Metabolic oxidative stress elicited by the copper(II) complex [cu(isaepy)2] triggers apoptosis in SH-SY5Y cells through the induction of the AMP-activated protein kinase/p38MAPK/p53 signalling axis: evidence for a combined use with 3-bromopyruvate in neuroblastoma treatment. Biochem. J. 437, 443–453. doi: 10.1042/BJ20110510, PMID: 21548882

[ref24] Food and Nutrition Board, Institute of Medicine (2001). Dietary reference intakes: Vitamin a, vitamin K, arsenic, boron, chromium, copper, iodine, iron, manganese, molybdenum, nickel, silicon, vanadium, and zinc. Washington, DC: National Academy Press. 25057538

[ref25] Frey B. N. Andreazza A. C. Kunz M. Gomes F. A. Quevedo J. Salvador M.. (2007). Increased oxidative stress and DNA damage in bipolar disorder: a twin-case report. Prog. Neuro-Psychopharmacol. Biol. Psychiatry 31, 283–285. doi: 10.1016/j.pnpbp.2006.06.011, PMID: 16859818

[ref26] Ge E. J. Bush A. I. Casini A. Cobine P. A. Cross J. R. DeNicola G. M.. (2022). Connecting copper and cancer: from transition metal signalling to metalloplasia. Nat. Rev. Cancer 22, 102–113. doi: 10.1038/s41568-021-00417-2, PMID: 34764459 PMC8810673

[ref27] Gernand A. D. Schulze K. J. Stewart C. P. West K. P. Christian P. (2016). Micronutrient deficiencies in pregnancy worldwide: health effects and prevention. Nat. Rev. Endocrinol. 12, 274–289. doi: 10.1038/nrendo.2016.37 27032981 PMC4927329

[ref28] Grubman A. White A. R. (2014). Copper as a key regulator of cell signalling pathways. Expert Rev. Mol. Med. 16:e11. doi: 10.1017/erm.2014.11, PMID: 24849048

[ref29] Ha J. H. Doguer C. Collins J. F. (2017). Consumption of a high-iron diet disrupts homeostatic regulation of intestinal copper absorption in adolescent mice. Am. J. Physiol. Gastrointest. Liver Physiol. 313, G353–G360. doi: 10.1152/ajpgi.00169.2017, PMID: 28619730 PMC5668571

[ref30] Habibi L. Tafakhori A. Hadiani R. Maserat-Mashhadi M. Kafrash Z. Torabi S.. (2017). Molecular changes in obese and depressive patients are similar to neurodegenerative disorders. Iran. J. Neurol. 16, 192–200. PMID: 29736225 PMC5937005

[ref31] Halliwell B. Gutteridge J. M. (1984). Oxygen toxicity, oxygen radicals, transition metals and disease. Biochem. J. 219, 1–14. doi: 10.1042/bj2190001, PMID: 6326753 PMC1153442

[ref32] Harvey L. J. Ashton K. Hooper L. Casgrain A. Fairweather-Tait S. J. (2009). Methods of assessment of copper status in humans: a systematic review. Am. J. Clin. Nutr. 89, 2009S–2024S. doi: 10.3945/ajcn.2009.27230E, PMID: 19420093

[ref33] Horng Y. C. Cobine P. A. Maxfield A. B. Carr H. S. Winge D. R. (2004). Specific copper transfer from the Cox17 metallochaperone to both Sco1 and Cox11 in the assembly of yeast cytochrome C oxidase. J. Biol. Chem. 279, 35334–35340. doi: 10.1074/jbc.M404747200 15199057

[ref34] Institute of Health Metrics and Evaluation (2023). Global health data exchange (GHDx). Available at: https://vizhub.healthdata.org/gbd-results/ (Accessed March 4, 2023).

[ref35] Institute of Medicine (US) Panel on Micronutrients (2001). Dietary reference intakes for vitamin a, vitamin K, arsenic, boron, chromium, copper, iodine, iron, manganese, molybdenum, nickel, silicon, vanadium, and zinc. Washington, DC: National Academies Press. 25057538

[ref36] Islam M. R. Islam M. R. Shalahuddin Qusar M. M. A. Islam M. S. Kabir M. H. Mustafizur Rahman G. K. M.. (2018). Alterations of serum macro-minerals and trace elements are associated with major depressive disorder: a case-control study. BMC Psychiatry 18:94. doi: 10.1186/s12888-018-1685-z 29631563 PMC5891975

[ref37] Jian Z. Guo H. Liu H. Cui H. Fang J. Zuo Z.. (2020). Oxidative stress, apoptosis and inflammatory responses involved in copper-induced pulmonary toxicity in mice. Aging 12, 16867–16886. doi: 10.18632/aging.103585, PMID: 32952128 PMC7521514

[ref38] Kessler R. C. (2003). Epidemiology of women and depression. J. Affect. Disord. 74, 5–13. doi: 10.1016/s0165-0327(02)00426-3 12646294

[ref39] Kim T. H. Choi J. Y. Lee H. H. Park Y. (2015). Associations between dietary pattern and depression in Korean adolescent girls. J. Pediatr. Adolesc. Gynecol. 28, 533–537. doi: 10.1016/j.jpag.2015.04.005, PMID: 26324576

[ref40] Kim H. Jeon B. T. Kim I. M. Bennett S. J. Lorch C. M. Viana M. P.. (2020). Sestrin2 phosphorylation by ULK1 induces autophagic degradation of mitochondria damaged by copper-induced oxidative stress. Int. J. Mol. Sci. 21:6130. doi: 10.3390/ijms21176130, PMID: 32854424 PMC7504119

[ref41] Klevay L. M. (2001). Iron overload can induce mild copper deficiency. J. Trace Elem. Med. Biol. 14, 237–240. doi: 10.1016/S0946-672X(01)80009-2 11396784

[ref42] Klevay L. M. (2016). IHD from copper deficiency: a unified theory. Nutr. Res. Rev. 29, 172–179. doi: 10.1017/S0954422416000093, PMID: 27350652

[ref43] Kouadri A. Cormenier J. Gemy K. Macari L. Charbonnier P. Richaud P.. (2021). Copper-associated oxidative stress contributes to cellular inflammatory responses in cystic fibrosis. Biomedicine 9:329. doi: 10.3390/biomedicines9040329, PMID: 33805052 PMC8064106

[ref44] Kumar J. Sathua K. B. Flora S. J. S. (2019). Chronic copper exposure elicit neurotoxic responses in rat brain: assessment of 8-hydroxy-2-deoxyguanosine activity, oxidative stress and neurobehavioral parameters. Cell. Mol. Biol. 65, 27–35. doi: 10.14715/cmb/2019.65.1.5, PMID: 30782290

[ref45] La Fontaine S. Mercer J. F. (2007). Trafficking of the copper-ATPases, ATP7A and ATP7B: role in copper homeostasis. Arch. Biochem. Biophys. 463, 149–167. doi: 10.1016/j.abb.2007.04.021, PMID: 17531189

[ref46] Lai J. S. Hiles S. Bisquera A. Hure A. J. McEvoy M. Attia J. (2014). A systematic review and meta-analysis of dietary patterns and depression in community-dwelling adults. Am. J. Clin. Nutr. 99, 181–197. doi: 10.3945/ajcn.113.069880, PMID: 24196402

[ref47] Lai C. C. Huang W. H. Klevay L. M. Gunning W. T. III Chiu T. H. (1996). Antioxidant enzyme gene transcription in copper-deficient rat liver. Free Radic. Biol. Med. 21, 233–240. doi: 10.1016/0891-5849(96)00029-9, PMID: 8818639

[ref48] Lee S. Y. Lee S. J. Han C. Patkar A. A. Masand P. S. Pae C. U. (2013). Oxidative/nitrosative stress and antidepressants: targets for novel antidepressants. Prog. Neuropsychopharmacol. Biol. Psychiatry 46, 224–235. doi: 10.1016/j.pnpbp.2012.09.008, PMID: 23022673

[ref49] Li Z. Li B. Song X. Zhang D. (2017). Dietary zinc and iron intake and risk of depression: a meta-analysis. Psychiatry Res. 251, 41–47. doi: 10.1016/j.psychres.2017.02.006, PMID: 28189077

[ref50] Li Q. Liao J. Zhang K. Hu Z. Zhang H. Han Q.. (2022). Toxicological mechanism of large amount of copper supplementation: effects on endoplasmic reticulum stress and mitochondria-mediated apoptosis by Nrf2/HO-1 pathway-induced oxidative stress in the porcine myocardium. J. Inorg. Biochem. 230:111750. doi: 10.1016/j.jinorgbio.2022.111750, PMID: 35151098

[ref51] Li Z. Wang W. Xin X. Song X. Zhang D. (2018). Association of total zinc, iron, copper and selenium intakes with depression in the US adults. J. Affect. Disord. 228, 68–74. doi: 10.1016/j.jad.2017.12.004, PMID: 29232566

[ref52] Liang Z. D. Tsai W. B. Lee M. Y. Savaraj N. Kuo M. T. (2012). Specificity protein 1 (sp1) oscillation is involved in copper homeostasis maintenance by regulating human high-affinity copper transporter 1 expression. Mol. Pharmacol. 81, 455–464. doi: 10.1124/mol.111.076422, PMID: 22172574 PMC3286298

[ref53] Liu T. Zhong S. Liao X. Chen J. He T. Lai S.. (2015). A meta-analysis of oxidative stress markers in depression. PLoS One 10:e0138904. doi: 10.1371/journal.pone.0138904, PMID: 26445247 PMC4596519

[ref54] Lu Q. Zhang Y. Zhao C. Zhang H. Pu Y. Yin L. (2022). Copper induces oxidative stress and apoptosis of hippocampal neuron via pCREB/BDNF/ and Nrf2/HO-1/NQO1 pathway. J. Appl. Toxicol. 42, 694–705. doi: 10.1002/jat.4252, PMID: 34676557

[ref55] Lutsenko S. Barnes N. L. Bartee M. Y. Dmitriev O. Y. (2007). Function and regulation of human copper-transporting ATPases. Physiol. Rev. 87, 1011–1046. doi: 10.1152/physrev.00004.2006 17615395

[ref56] Ma J. Betts N. M. (2000). Zinc and copper intakes and their major food sources for older adults in the 1994-96 continuing survey of food intakes by individuals (CSFII). J. Nutr. 130, 2838–2843. doi: 10.1093/jn/130.11.2838, PMID: 11053529

[ref57] Ma D. F. Zhang Y. M. Wang P. Y. Yang T. T. Tuo Y. Sheng Q. H. (2014). Analysis for the blood mineral content of children aged 3 to 12 years in 7 cities and 2 towns in China. Beijing Da Xue Xue Bao 46, 379–382. 24943014

[ref58] Maes M. Meltzer H. Y. Bosmans E. Bergmans R. Vandoolaeghe E. Ranjan R.. (1995). Increased plasma concentrations of interleukin-6, soluble interleukin-6, soluble interleukin-2 and transferrin receptor in major depression. J. Affect. Disord. 34, 301–309. doi: 10.1016/0165-0327(95)00028-l 8550956

[ref59] Maes M. Vandoolaeghe E. Neels H. Demedts P. Wauters A. Meltzer H. Y.. (1997). Lower serum zinc in major depression is a sensitive marker of treatment resistance and of the immune/inflammatory response in that illness. Biol. Psychiatry 42, 349–358. doi: 10.1016/S0006-3223(96)00365-4, PMID: 9276075

[ref60] Marx W. Moseley G. Berk M. Jacka F. (2017). Nutritional psychiatry: the present state of the evidence. Proc. Nutr. Soc. 76, 427–436. doi: 10.1017/S0029665117002026 28942748

[ref61] Mauskopf J. A. Simon G. E. Kalsekar A. Nimsch C. Dunayevich E. Cameron A. (2009). Nonresponse, partial response, and failure to achieve remission: humanistic and cost burden in major depressive disorder. Depress. Anxiety 26, 83–97. doi: 10.1002/da.20505, PMID: 18833573

[ref62] Mercer J. F. (1998). Menkes syndrome and animal models. Am. J. Clin. Nutr. 67, 1022S–1028S. doi: 10.1093/ajcn/67.5.1022S 9587146

[ref63] Moriarty A. S. Castleton J. Gilbody S. McMillan D. Ali S. Riley R. D.. (2020). Predicting and preventing relapse of depression in primary care. Br. J. Gen. Pract. 70, 54–55. doi: 10.3399/bjgp20X707753, PMID: 32001454 PMC7018432

[ref64] Mravunac M. Szymlek-Gay E. A. Daly R. M. Roberts B. R. Formica M. Gianoudis J.. (2019). Greater circulating copper concentrations and copper/zinc ratios are associated with lower psychological distress, but not cognitive performance, in a sample of Australian older adults. Nutrients 11:2503. doi: 10.3390/nu11102503, PMID: 31627408 PMC6836146

[ref65] Munakata M. Sakamoto O. Kitamura T. Ishitobi M. Yokoyama H. Haginoya K.. (2005). The effects of copper-histidine therapy on brain metabolism in a patient with Menkes disease: a proton magnetic resonance spectroscopic study. Brain and Development 27, 297–300. doi: 10.1016/j.braindev.2004.08.002, PMID: 15862194

[ref66] Nakamura M. Miura A. Nagahata T. Shibata Y. Okada E. Ojima T. (2019). Low zinc, copper, and manganese intake is associated with depression and anxiety symptoms in the Japanese working population: findings from the eating habit and well-being study. Nutrients 11:847. doi: 10.3390/nu11040847 30991676 PMC6521019

[ref67] Narang R. L. Gupta K. R. Narang A. P. Singh R. (1991). Levels of copper and zinc in depression. Indian J. Physiol. Pharmacol. 35, 272–274. PMID: 1812105

[ref68] National Academy of Sciences. (2000). Copper in drinking water. Prepared by the Board of Environmental Studies and Toxicology, Commission on Life Sciences, National Research Council. Washington, DC: National Academy Press.

[ref69] Ni M. You Y. Chen J. Zhang L. (2018). Copper in depressive disorder: a systematic review and meta-analysis of observational studies. Psychiatry Res. 267, 506–515. doi: 10.1016/j.psychres, PMID: 29980131

[ref70] Ohgami R. S. Campagna D. R. McDonald A. Fleming M. D. (2006). The Steap proteins are metalloreductases. Blood 108, 1388–1394. doi: 10.1182/blood-2006-02-003681, PMID: 16609065 PMC1785011

[ref71] Ozcelik D. Uzun H. (2009). Copper intoxication; antioxidant defenses and oxidative damage in rat brain. Biol. Trace Elem. Res. 127, 45–52. doi: 10.1007/s12011-008-8219-3 18784908

[ref72] Pereira T. C. Campos M. M. Bogo M. R. (2016). Copper toxicology, oxidative stress and inflammation using zebrafish as experimental model. J. Appl. Toxicol. 36, 876–885. doi: 10.1002/jat.3303, PMID: 26888422

[ref73] Pizzino G. Irrera N. Cucinotta M. Pallio G. Mannino F. Arcoraci V.. (2017). Oxidative stress: harms and benefits for human health. Oxidative Med. Cell. Longev. 2017:8416763. doi: 10.1155/2017/8416763, PMID: 28819546 PMC5551541

[ref74] Pyaskowit J. W. Prohaska J. R. (2008). Multiple mechanisms account for lower plasma iron in young copper deficient rats. Biometals 21, 343–352. doi: 10.1007/s10534-007-9123-6, PMID: 18038202 PMC2701467

[ref75] Pyatskowit J. W. Prohask J. R. (2008). Iron injection restores brain iron and hemoglobin deficits in perinatal copper-deficient rats. J. Nutr. 138, 1880–1886. doi: 10.1093/jn/138.10.1880, PMID: 18806096

[ref76] Reeves P. G. Demars L. C. (2005). Repletion of copper-deficient rats with dietary copper restores duodenal hephaestin protein and iron absorption. Exp. Biol. Med. 230, 320–325. doi: 10.1177/153537020523000505, PMID: 15855298

[ref77] Reeves P. G. Demars L. C. (2006). Signs of iron deficiency in copper-deficient rats are not affected by iron supplements administered by diet or by injection. J. Nutr. Biochem. 17, 635–642. doi: 10.1016/j.jnutbio.2006.04.004 16781861

[ref9001] Rihel J. (2018). Copper on the brain. Nat. Chem. Biol. 14, 638–639. doi: 10.1038/s41589-018-0089-1, PMID: 29915234

[ref78] Ruiz N. A. L. Del Ángel D. S. Brizuela N. O. Peraza A. V. Olguín H. J. Soto M. P.. (2022). Inflammatory process and immune system in major depressive disorder. Int. J. Neuropsychopharmacol. 25, 46–53. doi: 10.1093/ijnp/pyab072, PMID: 34724041 PMC8756095

[ref79] Ruizs L. M. Libedinsky A. Elorza A. A. (2021). Role of copper on mitochondrial function and metabolism. Front. Mol. Biosci. 8:711227. doi: 10.3389/fmolb.2021.711227, PMID: 34504870 PMC8421569

[ref80] Russo A. J. (2011). Analysis of plasma zinc and copper concentration, and perceived symptoms, in individuals with depression, post zinc and anti-oxidant therapy. Nutr. Metab. Insights. 4, 19–27. doi: 10.4137/NMI.S6760, PMID: 23946658 PMC3738484

[ref81] Salehi-Abargouei A. Esmaillzadeh A. Azadbakht L. Keshteli A. H. Afshar H. Feizi A.. (2019). Do patterns of nutrient intake predict self-reported anxiety, depression and psychological distress in adults? SEPAHAN study. Clin. Nutr. 38, 940–947. doi: 10.1016/j.clnu.2018.02.002, PMID: 29503058

[ref82] Salustri C. Squitti R. Zappasodi F. Ventriglia M. Bevacqua M. G. Fontana M.. (2010). Oxidative stress and brain glutamate-mediated excitability in depressed patients. J. Affect. Disord. 127, 321–325. doi: 10.1016/j.jad.2010.05.012 20547423

[ref83] Sharma S. K. Sood S. Sharma A. Gupta I. D. (2014). Estimation of serum zinc and copper levels patients with schizophrenia: a preliminary study. Sri. Lanka. J. Psyc. 5, 14–17. doi: 10.4038/sljpsyc.v5i1.7076

[ref84] Shusharina N. Yukhnenko D. Botman S. Sapunov V. Savinov V. Kamyshov G.. (2023). Modern methods of diagnostics and treatment of neurodegenerative diseases and depression. Diagnostics 13:573. doi: 10.3390/diagnostics13030573, PMID: 36766678 PMC9914271

[ref85] Siwek M. Styczeń K. Sowa-Kućma M. Dudek D. Reczyński W. Szewczyk B.. (2017). The serum concentration of copper in bipolar disorder. Psychiatr. Pol. 51, 469–481. doi: 10.12740/PP/OnlineFirst/65250, PMID: 28866717

[ref86] Styczeń K. Sowa-Kućma M. Siwek M. Dudek D. Reczyński W. Misztak P.. (2016). Study of the serum copper levels in patients with major depressive disorder. Biol. Trace Elem. Res. 174, 287–293. doi: 10.1007/s12011-016-0720-5, PMID: 27147437 PMC5090008

[ref87] Sun X. Li J. Zhao H. Wang Y. Liu J. Shao Y.. (2018). Synergistic effect of copper and arsenic upon oxidative stress, inflammation and autophagy alterations in brain tissues of Gallus gallus. J. Inorg. Biochem. 178, 54–62. doi: 10.1016/j.jinorgbio.2017.10.006, PMID: 29054015

[ref88] Thi Thu Nguyen T. Miyagi S. Tsujiguchi H. Kambayashi Y. Hara A. Nakamura H.. (2019). Association between lower intake of minerals and depressive symptoms among elderly Japanese women but not men: findings from Shika study. Nutrients 11:389. doi: 10.3390/nu11020389, PMID: 30781841 PMC6412241

[ref89] Turnlund J. R. (2000). “Copper status and metabolism studied with isotopic tracers” in Advances in isotope methods for the analysis of trace elements in man. eds. Jackson M. Lowe N. (Boca Raton: CRC Press)

[ref90] Twayej A. J. Al-Hakeim H. K. Al-Dujaili A. H. Maes M. (2020). Lowered zinc and copper levels in drug-naïve patients with major depression: effects of antidepressants, ketoprofen and immune activation. World J. Biol. Psychiatry 21, 127–138. doi: 10.1080/15622975.2019.1612090, PMID: 31062629

[ref91] Uauy R. Olivares M. Gonzalez M. (1998). Essentiality of copper in humans. Am. J. Clin. Nutr. 67, 952S–959S. doi: 10.1093/ajcn/67.5.952S 9587135

[ref92] Ullas Kamath S. Chaturvedi A. Bhaskar Yerrapragada D. Kundapura N. Amin N. Devaramane V. (2019). Increased levels of acetylcholinesterase, paraoxonase 1, and copper in patients with moderate depression-a preliminary study. Rep. Biochem. Mol. Biol. 7, 174–180. PMID: 30805397 PMC6374063

[ref93] Uriu-Adams J. Y. Keen C. L. (2005). Copper, oxidative stress, and human health. Mol. Asp. Med. 26, 268–298. doi: 10.1016/j.mam.2005.07.015, PMID: 16112185

[ref94] Wang T. Xiang P. Ha J. H. Wang X. Doguer C. Flores S. R. L.. (2018). Copper supplementation reverses dietary iron overload-induced pathologies in mice. J. Nutr. Biochem. 59, 56–63. doi: 10.1016/j.jnutbio.2018.05.006, PMID: 29960117 PMC6467079

[ref95] Wang C. J. Yang T. F. Wang G. S. Zhao Y. Y. Yang L. J. Bi B. N. (2018). Association between dietary patterns and depressive symptoms among middle-aged adults in China in 2016-2017. Psychiatry Res. 260, 123–129. doi: 10.1016/j.psychres.2017.11.052, PMID: 29182923

[ref96] Wang Y. Zhao H. Shao Y. Liu J. Li J. Luo L.. (2018). Copper or/and arsenic induces autophagy by oxidative stress-related PI3K/AKT/mTOR pathways and cascaded mitochondrial fission in chicken skeletal muscle. J. Inorg. Biochem. 188, 1–8. doi: 10.1016/j.jinorgbio.2018.08.001 30096535

[ref97] West E. C. Prohaska J. R. (2004). Cu,Zn-superoxide dismutase is lower and copper chaperone CCS is higher in erythrocytes of copper-deficient rats and mice. Exp. Biol. Med. 229, 756–764. doi: 10.1177/153537020422900807 15337829

[ref98] Winge D. R. Mehra R. K. (1990). Host defenses against copper toxicity. Int. Rev. Exp. Pathol. 31, 47–83. doi: 10.1016/b978-0-12-364931-7.50007-0 2292474

[ref99] World Health Organization. (2023a). Depressive disorder (depression). Available at: https://www.who.int/news-room/fact-sheets/detail/depression (Accessed March 31, 2023).

[ref100] World Health Organization. (2023b). Depression. Available at: https://www.who.int/news-room/fact-sheets/detail/depression (Accessed January13, 2023).

[ref101] Wu H. R. Li Q. Q. Gao R. Tang S. Y. Zhang K. F. Zhao J. F. (2022). BMI modifies the association between depression symptoms and serum copper levels. Biol. Trace Elem. Res. 201, 4216–4229. doi: 10.1007/s12011-022-03505-y 36437432

[ref102] Xia Y. Wang N. Yu B. Zhang Q. Liu L. Meng G.. (2017). Dietary patterns are associated with depressive symptoms among Chinese adults: a case-control study with propensity score matching. Eur. J. Nutr. 56, 2577–2587. doi: 10.1007/s00394-016-1293-y 27543189

[ref103] Xie J. He X. Fang H. Liao S. Liu Y. Tian L.. (2020). Identification of heme oxygenase-1 from golden pompano (*Trachinotus ovatus*) and response of Nrf2/HO-1 signaling pathway to copper-induced oxidative stress. Chemosphere 253:126654. doi: 10.1016/j.chemosphere.2020.126654, PMID: 32464761

[ref104] Xu J. He K. Zhang K. Yang C. Nie L. Dan D.. (2021). Low-dose copper exposure exacerbates depression-like behavior in ApoE4 transgenic mice. Oxidative Med. Cell. Longev. 2021:6634181. doi: 10.1155/2021/6634181, PMID: 33833851 PMC8018851

[ref105] Yang F. Liao J. Yu W. Pei R. Qiao N. Han Q.. (2020). Copper induces oxidative stress with triggered NF-κB pathway leading to inflammatory responses in immune organs of chicken. Ecotoxicol. Environ. Saf. 200:110715. doi: 10.1016/j.ecoenv.2020.110715 32450432

[ref106] Zhang F. Zheng W. Guo R. Yao W. (2017). Effect of dietary copper level on the gut microbiota and its correlation with serum inflammatory cytokines in Sprague-Dawley rats. J. Microbiol. 55, 694–702. doi: 10.1007/s12275-017-6627-9, PMID: 28865069

[ref107] Zhong C. C. Zhao T. Hogstrand C. Chen F. Song C. C. Luo Z. (2022). Copper (cu) induced changes of lipid metabolism through oxidative stress-mediated autophagy and Nrf2/PPARγ pathways. J. Nutr. Biochem. 100:108883. doi: 10.1016/j.jnutbio.2021.108883, PMID: 34653601

[ref108] Zhong W. Zhu H. Sheng F. Tian Y. Zhou J. Chen Y.. (2014). Activation of the MAPK11/12/13/14 (p38 MAPK) pathway regulates the transcription of autophagy genes in response to oxidative stress induced by a novel copper complex in HeLa cells. Autophagy 10, 1285–1300. doi: 10.4161/auto.28789 24905917 PMC4203553

[ref109] Zou L. Cheng G. Xu C. Liu H. Wang Y. Li N.. (2021). Copper nanoparticles induce oxidative stress via the heme oxygenase 1 signaling pathway in vitro studies. Int. J. Nanomedicine 16, 1565–1573. doi: 10.2147/IJN.S292319, PMID: 33664571 PMC7924257

